# A mechanism for overcoming P-glycoprotein-mediated drug resistance: novel combination therapy that releases stored doxorubicin from lysosomes via lysosomal permeabilization using Dp44mT or DpC

**DOI:** 10.1038/cddis.2016.381

**Published:** 2016-12-01

**Authors:** Nicole A Seebacher, Des R Richardson, Patric J Jansson

**Affiliations:** 1 Molecular Pharmacology and Pathology Program, Department of Pathology, University of Sydney, Blackburn Building (D06), Sydney, New South Wales, Australia

## Abstract

The intracellular distribution of a drug can cause significant variability in both activity and selectivity. Herein, we investigate the mechanism by which the anti-cancer agents, di-2-pyridylketone 4,4-dimethyl-3-thiosemicarbazone (Dp44mT) and the clinically trialed, di-2-pyridylketone 4-cyclohexyl-4-methyl-3-thiosemicarbazone (DpC), re-instate the efficacy of doxorubicin (DOX), in drug-resistant P-glycoprotein (Pgp)-expressing cells. Both Dp44mT and DpC potently target and kill Pgp-expressing tumors, while DOX effectively kills non-Pgp-expressing cancers. Thus, the combination of these agents should be considered as an effective rationalized therapy for potently treating advanced and resistant tumors that are often heterogeneous in terms of Pgp-expression. These studies demonstrate that both Dp44mT and DpC are transported into lysosomes via Pgp transport activity, where they induce lysosomal-membrane permeabilization to release DOX trapped within lysosomes. This novel strategy of loading lysosomes with DOX, followed by permeabilization with Dp44mT or DpC, results in the relocalization of stored DOX from its lysosomal 'safe house' to its nuclear targets, markedly enhancing cellular toxicity against resistant tumor cells. Notably, the combination of Dp44mT or DpC with DOX showed a very high level of synergism in multiple Pgp-expressing cell types, for example, cervical, breast and colorectal cancer cells. These studies revealed that the level of drug synergy was proportional to Pgp activity. Interestingly, synergism was ablated by inhibiting Pgp using the pharmacological inhibitor, Elacridar, or by inhibiting Pgp-expression using Pgp-silencing, demonstrating the importance of Pgp in the synergistic interaction. Furthermore, lysosomal-membrane stabilization inhibited the relocalization of DOX from lysosomes to the nucleus upon combination with Dp44mT or DpC, preventing synergism. This latter observation demonstrated the importance of lysosomal-membrane permeabilization to the synergistic interaction between these agents. The synergistic and potent anti-tumor efficacy observed between DOX and thiosemicarbazones represents a promising treatment combination for advanced cancers, which are heterogeneous and composed of non-Pgp- and Pgp-expressing tumor cells.

A major limitation of the cytotoxic chemotherapeutic, Doxorubicin (DOX; Figure 1ai), is resistance caused by drug-resistance pumps, for example, P-glycoprotein (Pgp).^1, 2^ This has been attributed to both Pgp-mediated drug efflux through the plasma membrane^3^ and also due to increased Pgp-mediated drug trapping within lysosomal drug 'safe houses' (Figure 1aii).^4^ The latter has been shown to be due to Pgp being topological inverted during the endocytic process to face inward into lysosomes leading to lysosomal-loading of Pgp substrates (Figure 1aii).^4, 5, 6^ In this case, DOX is stored in lysosomes due to it becoming protonated in the acidic pH of this organelle, preventing its distribution to its major targets in the nucleus, leading to drug resistance.^4^

Interestingly, a novel class of anti-cancer agents, the di-2-pyridylketone thiosemicarbazone (DpT) analogs, has demonstrated potent and selective activity and the ability to overcome multidrug resistance by directly utilizing lysosomal Pgp-transport activity.^5, 6^ These agents include the first generation compound, di-2-pyridylketone 4,4-dimethyl-3-thiosemicarbazone (Dp44mT; Figure 1bi),^7, 8, 9^ and also the more selective second generation analog, di-2-pyridylketone 4-cyclohexyl-4-methyl-3-thiosemicarbazone (DpC; Figure 1bii),^10, 11^ which has entered clinical trials (NCT02688101).^12^ These agents are Pgp substrates and are effluxed out of cells, but also transported into lysosomes by utilizing Pgp on the lysosomal membrane (Figure 1biii).^5, 6, 13^

Within lysosomes, thiosemicarbazones become trapped due to protonation at the acidic pH of the organelle.^5, 9, 14, 15^ These agents then bind copper and redox cycle to generate reactive oxygen species that induce lysosomal-membrane permeabilization (LMP), which results in apoptotic death, thereby overcoming Pgp-mediated resistance (Figure 1biii).^5, 9, 14, 15, 16^ Hence, this mechanism is opposite to DOX (Figure 1aii), which does not induce lysosomal permeabilization and remains securely trapped within lysosomes, inducing resistance. Within the lysosomal safe house, DOX is unable to reach its target, namely the nucleus, and thus, resistance to DOX occurs (Figure 1aii).^4^

Herein, we report that Dp44mT or DpC, in addition to their own pronounced anti-cancer effects via LMP and induction of apoptosis (Figure 1biii),^5, 6, 7, 9, 14^ also cause the release of stored DOX from lysosomes in highly drug-resistant Pgp-expressing cells. Both these potent cytotoxic effects result in synergism between these agents that markedly enhances antiproliferative efficacy against tumor cells hyper-expressing Pgp. Indeed, Pgp activity was a strong predictor of synergistic efficacy between these agents. These studies rationalize a novel mechanism for targeting drug-resistant cancers via the strategic implementation of drug combinations, which could improve treatment outcomes in Pgp-expressing tumors.

## Results

### Cellular Pgp-expression is increased by DOX, but decreased by Dp44mT and DpC

**Figure 1:**
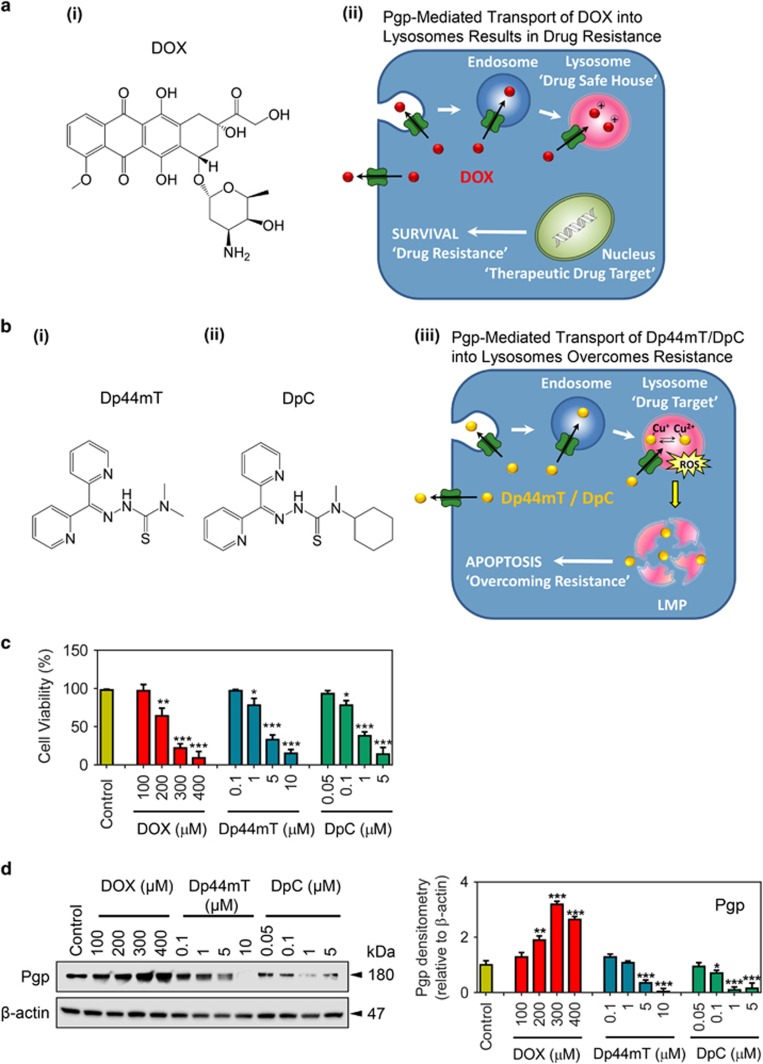
Pgp expression is altered following treatment of cancer cells with increasing concentrations of DOX or the thiosemicarbazones, Dp44mT and DpC. (**a**) (i) Line drawing of the structure of doxorubicin (DOX). (**a**) (ii) Schematic showing that DOX is effluxed out of cells as it is a substrate of the drug efflux pump, P-glycoprotein (Pgp),^4^ but can also be transported by Pgp into endosomes and lysosomes upon endocytosis.^4^ Storage of DOX in the lysosome leads to multidrug resistance (MDR) as DOX is sequestered away from its major target, the nucleus (so-called lysosomal 'safe-house' effect).^4^ (**b**) (i,ii) Line drawings of the structures of Dp44mT and DpC. (**b**) (iii) Schematic demonstrating that Pgp facilitates Dp44mT and DpC transport out of the cells and also into endosomes/lysosomes.^5, 6^ However, these agents overcome Pgp-mediated drug resistance by forming copper complexes that potently generate reactive oxygen species (ROS).^5, 6, 16^ The generation of ROS causes rapid lysosomal-membrane permeabilization (LMP), and apoptosis that leads to the death of the resistant cancer cell.^5, 6, 9^ Hence, the lysosome is a novel drug target that can be implemented against Pgp-expressing cancers by utilizing their high levels of lysosomal Pgp. (**c** and **d**) KBV1 (+Pgp) cells were treated with DOX (72 h/37 °C; 100–400 *μ*M), Dp44mT (24 h/37 °C; 0.1–10 *μ*M) or DpC (24 h/37 °C; 0.05–5 *μ*M) and assessed for (**c**) cell viability by trypan blue-staining and (**d**) Pgp protein expression by western blotting. *P<0.05, **P<0.01, ***P<0.001 relative to the control (no treatment). The western blot shown is a typical experiment of 3 performed. Densitometry is relative to *β*-actin and is mean±S.D. (three experiments)

Considering that Pgp regulates the activity of DOX,^3, 4^ Dp44mT^5^ and DpC,^5, 6^ it was important to assess how these drugs affect cellular viability and Pgp protein levels. The KBV1 cell type highly expressing Pgp was incubated with high DOX concentrations (100–400 *μ*M; 72 h/37 °C), considering their high resistance to this agent,^24^ and then viability and Pgp expression examined. A shorter 24 h/37 °C incubation was utilized for the highly cytotoxic thiosemicarbazones, Dp44mT and DpC,^5, 6, 7, 8, 9, 10, 11^ as longer times led to almost total cellular ablation. DOX, Dp44mT and DpC all significantly (*P<*0.001–0.05) decreased viability at concentrations ⩾200 *μ*M, ⩾1 *μ*M and ⩾0.1 *μ*M, respectively (Figure 1c).

Compared with untreated cells, DOX (200–400 *μ*M) significantly (*P<*0.001–0.01) increased Pgp expression by 1.9–3.2-fold (Figure 1d). In contrast, Dp44mT (5–10 *μ*M) and DpC (0.1–5 *μ*M) significantly (*P<*0.001–0.05) decreased Pgp by 1.5–30-fold (Figure 1d). These findings demonstrate that DOX increases Pgp, while Dp44mT and DpC decrease it. These observations could be explained by DOX killing cells lacking Pgp and selecting for cells expressing this drug pump. In contrast, Dp44mT and DpC selectively target Pgp-expressing cells over non-Pgp-expressing cells,^5, 6^ demonstrating the advantage of these thiosemicarbazones over DOX.

### Pgp activity enhances synergism between DOX and thiosemicarbazones

**Figure 2:**
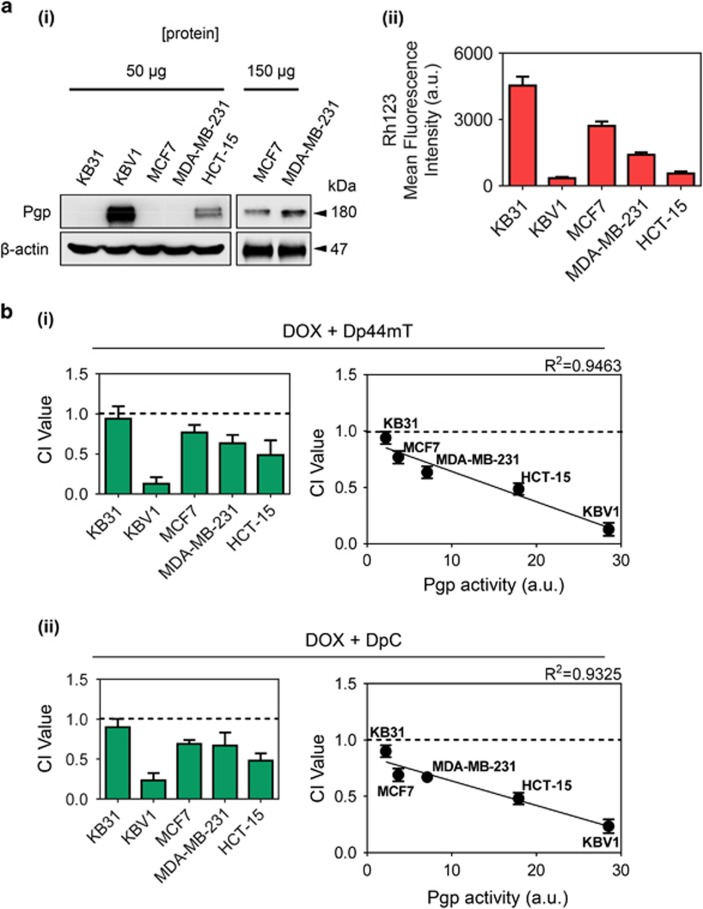
Pgp activity enhances drug synergy between DOX and the thiosemicarbazones, Dp44mT and DpC. KB31, KBV1, MCF7, MDA-MB-231 and HCT-15 cell lines were assessed for: (**a**) (i) Pgp expression by western blotting and (ii) cellular Rh123 retention measured by flow cytometry. (**b**) Combination index (CI) values of the drug combinations between (i) DOX (72 h/37 °C) and Dp44mT (24 h/37 °C), (ii) DOX (72 h/37 °C) and DpC (24 h/37 °C), as measured by the Chou-Talalay method.^25, 29^ CI>1 Antagonism, CI=1 Additive, CI<1 Synergistic. The correlation between the CI values (**b**) (i), (ii) and the Pgp activity (**a**) (ii) was plotted using linear regression. The western shown is a typical experiment of 3 performed. Results are shown as mean±S.D. (three experiments)

Within tumors there exists a heterogeneous population of drug-resistant and drug-sensitive cells.^28^ Considering that DOX is selectively cytotoxic to non-Pgp-expressing cells,^3, 4, 5^ resulting in the selection of Pgp-expression (Figure 1d), and that Dp44mT and DpC are more cytotoxic to highly Pgp-expressing cells (Figures 1c and d),^5, 6^ the combination of Dp44mT or DpC, and DOX may lead to synergism. Studies investigating synergism were then performed in a range of cell types with varying Pgp-expression, as determined by western blotting (Figure 2ai). Notably, KBV1 cells had the highest levels of Pgp-expression, while KB31 cells demonstrated extremely low Pgp expression that was not detected by westerns (Figure 2ai). In fact, the very low Pgp levels in KB31 cells could only be detected by more sensitive flow cytometry.^24^ Surface Pgp function was then assessed in these cell types by cellular retention of the fluorescent Pgp substrate, Rh123 (Figure 2aii). As expected, Rh123 retention was inversely correlated with Pgp expression, with cells highly expressing Pgp retaining low Rh123 levels and vice versa (Figure 2aii).

To mimic the development of resistance, and because Pgp-expression is important for Dp44mT and DpC cytotoxicity,^5, 6^ all cell types were pre-treated with DOX for 48 h/37 °C to select for populations with increased Pgp (Figure 1d). Then Dp44mT or DpC were added with DOX for the remaining 24 h/37 °C, resulting in a total incubation of 72 h/37 °C (Figure 2bi). To investigate drug combination effects, the Chou-Talalay method was used,^25, 29^ which results in the calculation of combination index (CI) values, where CI>1 denotes antagonism, CI=1 signifies an additive relationship and CI<1 indicates synergism.

Drug synergy between DOX and Dp44mT was greatest in Pgp hyper-expressing KBV1 cells (CI: 0.13; Figure 2bi). In all other cell types where Pgp expression was observed (Figure 2ai), the drug interactions were also synergistic (CI: 0.48–0.76; Figure 2bi). In contrast, in KB31 cells that express extremely low Pgp levels,^24^ the effect was additive (CI: 0.94; Figure 2bi). When the CI values were plotted against the Pgp-substrate transport activity of the cell types (Figure 2aii), a strong inverse correlation (*R*^2^=0.9463) was found between Pgp activity and CI value (Figure 2bi). That is, higher Pgp activity was correlated with greater synergy (i.e., lower CI value). Similarly, when DOX was combined with DpC, drug synergy was also inversely correlated to the Pgp activity of the cell type (*R*^2^=0.9325; Figure 2bii). Together, these results indicate the combination of DOX with Dp44mT or DpC is highly synergistic when cells have greater Pgp activity.

### Drug synergy between DOX and thiosemicarbazones is Pgp dependent

**Figure 3:**
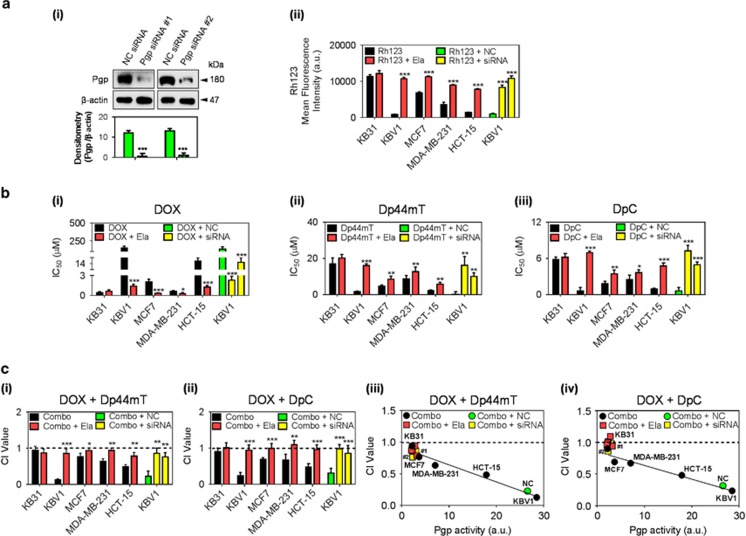
Inhibition of Pgp activity using the inhibitor, Elacridar (Ela), or Pgp expression using two Pgp siRNAs prevents synergy between DOX and Dp44mT or DpC. Western blotting was used to assess Pgp expression in: (**a**) (i) KBV1 (+Pgp) cells incubated for 72 h/37 °C with two different Pgp siRNAs or a negative control siRNA (NC siRNA); (ii) Pgp activity in all cell lines assessed by retention of the Pgp substrate, Rh123 (10 *μ*M; 30 min/37 °C). (**b**) Cytotoxicity assays with the treatments: (i) DOX (72 h/37 °C), (ii) Dp44mT (24 h/37 °C) and (iii) DpC (24 h/37 °C), in the absence or presence of Ela (0.2 *μ*M) or Pgp siRNA. (**c**) Combination index (CI) values of the drug combinations between: (i) DOX (72 h/37 °C) and Dp44mT (24 h/37 °C); or (ii) DOX (72 h/37 °C) and DpC (24 h/37 °C), as measured by the Chou-Talalay method.^25, 29^ The correlation between the CI values (**c**iii,iv) and the cellular Pgp activity (**a**ii) was plotted using linear regression. *P<0.05, **P<0.01, ***P<0.001 relative to respective non-Ela- or NC-siRNA-treated control (black=Combo (no Ela); red=Combo+Ela; green=Combo+negative control (NC) siRNA; yellow=Combo+siRNA (Pgp siRNA)). Western blots are typical of three experiments. Results are shown as mean±S.D. (three experiments)

We next examined if Pgp was necessary for the synergistic interaction between DOX and the thiosemicarbazones. This was examined by inhibiting Pgp with the highly specific Pgp-activity inhibitor, Elacridar (Ela),^30^ or silencing Pgp using siRNAs. Initial studies demonstrated that, as expected, the Pgp-activity inhibitor, Ela (0.2 *μ*M/24 h/37 °C), did not significantly (*P*>0.05) inhibit Pgp expression in all cell types (not shown), nor did it alter lysosomal integrity.^6, 16^ In contrast, Pgp silencing, using two different siRNAs (Pgp siRNA #1 and #2), significantly (*P*<0.001) decreased Pgp expression in KBV1 cells relative to the negative control siRNA (NC siRNA; Figure 3ai). Importantly, either Ela or Pgp siRNA significantly (*P*<0.001) increased Rh123 retention in Pgp-expressing KBV1 cells (Figure 3aii).

To examine the effect of inhibiting or silencing Pgp on drug anti-tumor activity, cytotoxicity assays were then performed (Figures 3bi–biii). As expected, Ela significantly (*P<*0.001–0.05) sensitized all Pgp-expressing cell lines to DOX treatment, leading to a decrease in IC_50_ (Figure 3bi). This was most evident for KBV1 hyper-expressing Pgp cells, which showed the greatest resistance (highest IC_50_) to DOX under control conditions. Interestingly, KB31 cells, which were 354-fold more sensitive to DOX than KBV1 cells, demonstrated no significant (*P>*0.05) change in the sensitivity to DOX upon co-incubation with Ela (Figure 3bi). This effect was attributed to their extremely low Pgp-expression.^24^ Similarly to Ela, both Pgp siRNAs significantly (*P<*0.001) sensitized KBV1 cells to DOX (Figure 3bi). These results demonstrate Pgp is responsible for resistance to DOX.

In marked contrast, Ela or Pgp-silencing significantly (*P<*0.001–0.01) decreased the cytotoxicity of Dp44mT and DpC in KBV1 cells, resulting in an increased IC_50_ (Figures 3bii and biii). Notably, Ela also significantly (*P<*0.001–0.05) decreased the cytotoxicity of Dp44mT or DpC in MCF7, MDA-MB-231 and HCT-15 cells (Figures 3bii and biii). On the other hand, Ela did not have any significant effect on Dp44mT or DpC activity in KB31 cells that express extremely low Pgp levels^24^ (Figures 3bii and biii). Together, these results indicate Pgp is responsible for the increased cellular sensitivity to Dp44mT and DpC.

Addition of Ela to the combination of DOX and Dp44mT (Combo+Ela; Figure 3ci) caused a significant (*P<*0.001–0.05) increase in CI values (decreased synergy) in all Pgp-expressing cell types, but was not significantly (*P>*0.05) altered in KB31 cells. Similarly, in Pgp-expressing cell lines that demonstrated synergy between the combination of DOX and DpC, the combination became significantly (*P*<0.001–0.01) less synergistic in the presence of Ela (Combo+Ela; Figure 3cii). Moreover, Pgp silencing in KBV1 cells in the presence of the drug combination (Combo+siRNA) also significantly (*P*<0.001–0.01) reversed drug synergy between DOX and Dp44mT or DOX and DpC relative to the negative control (NC) siRNA (Combo+NC; Figures 3ci and cii).

The plots between the CI values (Figures 3ci and cii) and Pgp activity (Figure 3aii) in Figures 3ciii and civ indicate for each cell type (black symbols) the synergism for the Combo, namely: (1) DOX+Dp44mT (Figure 3ciii); (2) DOX+DpC (Figure 3civ); or (3) Combo with NC siRNA (Combo+NC; green symbol; Figures 3ciii and civ); or Combo with Pgp siRNA (Combo+siRNA; yellow symbol; Figure 3civ). A marked decrease in synergism for each of these cell types was demonstrated after the addition of either Ela to the Combo's (Combo+Ela; red symbols) or Pgp siRNA to the Combo (Combo+siRNA; yellow symbols; Figures 3ciii and civ).

Together, these data indicate that inhibiting Pgp activity or expression markedly decreases drug synergy between DOX and Dp44mT or DOX and DpC.

### DOX is re-distributed from lysosomes to the nucleus following lysosomal damage by thiosemicarbazones in Pgp-expressing cells

**Figure 4:**
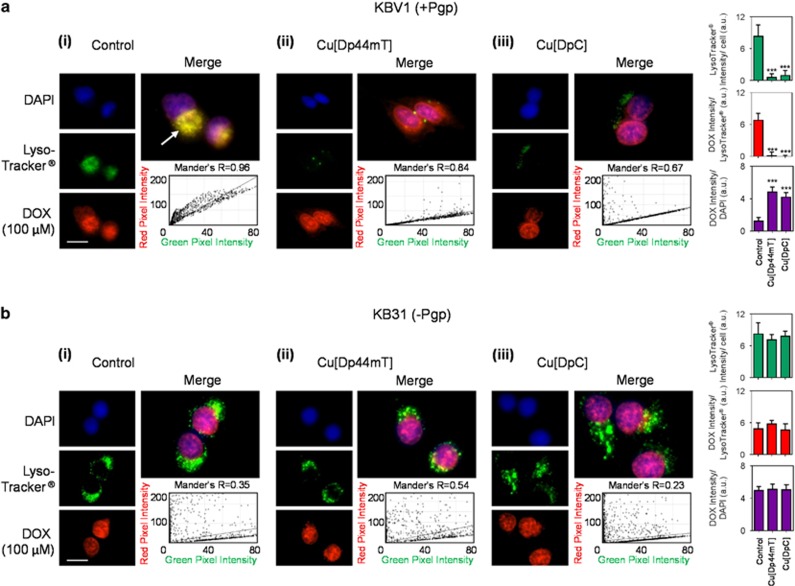
Co-localization of DOX with LysoTracker Green-stained lysosomes decreases with treatment with Dp44mT or DpC. Immunofluorescence microscopy images of: (**a**) KBV1 (+Pgp) and (**b**) KB31 cells (expressing extremely low Pgp levels)^24^ incubated with: (i) no treatment (Control); (ii) Cu[Dp44mT] (30 *μ*M); or (iii) Cu[DpC] (15 *μ*M) for 30 min/37 °C following staining with DOX (2 h/37 °C; 100 *μ*M) and LysoTracker Green (40 min/37 °C; 100 nM). The overlap between DOX and LysoTracker Green (yellow in the merge) is indicated by the Mander's overlap coefficient, (*R*). LysoTracker Green was quantified as fluorescence intensity/cell in arbitrary units (a.u.). DOX was quantified as fluorescence intensity co-localization with LysoTracker Green using ImageJ software. ***P<0.001, relative to Control (no treatment). Scale bar=10 *μ*m. Photographs are typical of three experiments. Quantitation is mean±S.D. (three experiments)

We next examined the mechanisms involved in the drug synergy between DOX and Dp44mT or DOX and DpC in Pgp-expressing cells (Figure 4). The major targets of DOX are considered to occur within the nucleus and include DNA intercalation and inhibition of topoisomerase.^31, 32^ Moreover, DOX toxicity is limited by expression of plasma membrane Pgp that leads to cellular efflux^4, 33^ and lysosomal Pgp that results in lysosomal-loading of DOX in the lysosomal 'safe house' (Figure 1aii).^4^

We hypothesized that LMP caused by Dp44mT or DpC could release DOX from lysosomes, which would overcome resistance by increasing nuclear targeting of DOX. Therefore, the distribution of DOX was examined following incubation with the thiosemicarbazones. The KBV1 and KB31 cell types were chosen for these studies due to their very high and very low Pgp levels, respectively^4, 6, 24^ (Figure 2ai). Copper-thiosemicarbazone complexes (i.e., Cu[Dp44mT] and Cu[DpC]) were used for these live cell-imaging experiments as they cause more rapid LMP than the ligand alone, while still using the same lysosomal targeting and disruption mechanism.^5, 6, 9^

DOX (100 *μ*M/2 h/37 °C) exhibits its own intrinsic fluorescence and showed a distinct cytosolic-staining pattern in KBV1 cells under control conditions, which overlapped with LysoTracker Green-stained lysosomes in the merge (yellow; Mander's overlap coefficient (*R*)=0.96; Figure 4ai). This result is consistent with DOX trapped within lysosomes.^4^ When DOX treatment was followed by co-incubation with either Cu[Dp44mT] (30 *μ*M/30 min/37 °C; Figure 4aii) or Cu[DpC] (15 *μ*M/30 min/37 °C; Figure 4aii), the cytosolic-staining pattern of LysoTracker Green largely disappeared, which is consistent with LMP.^34^ Consequently, the overlap of DOX with LysoTracker Green-stained lysosomes was significantly (*P<*0.001) decreased (Figures 4aii and aiii). Instead, DOX was now observed to markedly overlap with the DAPI-stained nucleus (*R*=0.98). These observations indicate Cu[Dp44mT] or Cu[DpC] induce LMP with the release of DOX from lysosomes and its subsequent entrance into nuclei.

In contrast to KBV1 Pgp-expressing cells, using KB31 cells that express extremely low Pgp levels,^6, 24^ under control conditions, DOX-staining overlapped with nuclear DAPI (*R*=0.94) and showed very minimal co-localization with LysoTracker Green-stained lysosomes (*R*=0.35; Figure 4bi). Hence, due to the lack of Pgp-transport into lysosomes of KB31 cells, there was no accumulation of DOX in this compartment. Furthermore, no damage to lysosomes was induced by Cu[Dp44mT] (Figure 4bii) or Cu[DpC] (Figure 4biii), there being no significant (*P>*0.05) change in LysoTracker Green staining. In this case, DOX remained in the nuclei overlapped with DAPI (DOX+Cu[Dp44mT], *R*=0.97; DOX+Cu[DpC], *R*=0.93; Figures 4bii and biii). Hence, in the absence of Pgp, the thiosemicarbazones were not transported into lysosomes to induce LMP, and thus, no DOX release occurred.

Together, these results in Figure 4 indicate that Pgp results in DOX accumulation in lysosomes and that subsequent thiosemicarbazone transport into lysosomes via Pgp induces LMP, and thus the redistribution of lysosomal-trapped DOX to nuclei.

### Cholesterol retention via incubation with U18666A prevents thiosemicarbazone-induced LMP, thereby preventing redistribution of DOX to the nucleus

To further investigate the importance of LMP in thiosemicarbazone-induced redistribution of DOX from lysosomes to nuclei, the cholesterol transport inhibitor, 3-*β*-[2-(diethyl-amino)ethoxy]androst-5-en-17-one (U18666A),^14, 35, 36^ was utilized. Notably, U18666A results in cholesterol retention in lysosomal membranes, which stabilizes lysosomes to oxidative damage.^14, 36, 37^ Therefore, U18666A was utilized to prevent Dp44mT or DpC-induced LMP. Initially, lysosomal integrity was examined using the classical lysosomotropic dye, acridine orange (AO).^38^ An increase in green cytosolic AO staining, corresponding with a decrease in red AO intensity in lysosomes, is consistent with a loss of lysosomal integrity.^39^

**Figure 5:**
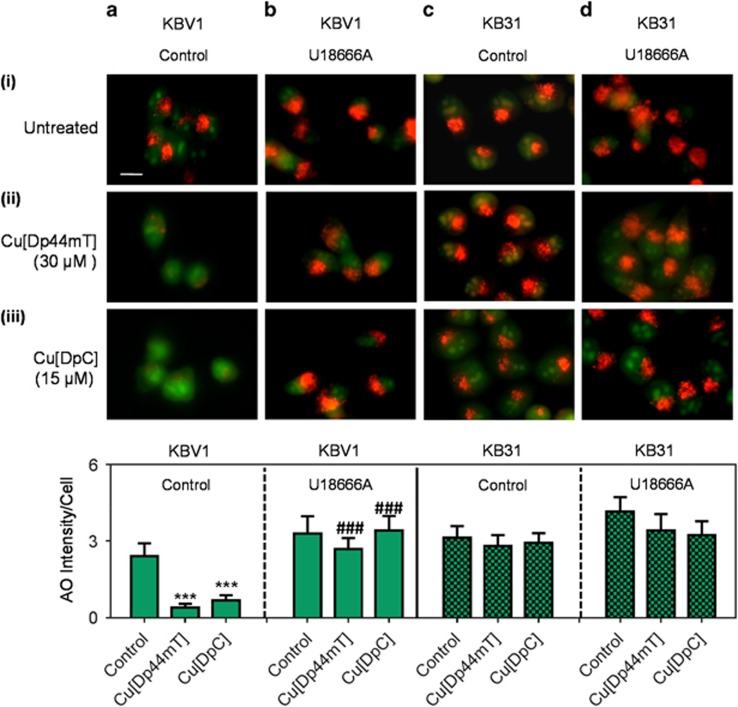
The cholesterol transport inhibitor, U18666A, prevents lysosomal-membrane permeabilization (LMP) following treatment with Dp44mT or DpC. Live cell immunofluorescence microscopy images using the following conditions: (**a**) KBV1 cells (+Pgp); (**b**) KBV1 cells with U18666A (2.3 *μ*g/ml); (**c**) KB31 cells (expressing extremely low Pgp levels)^24^ and (**d**) KB31 cells with U18666A (2.3 *μ*g/ml). Cells were incubated for 30 min/37 °C with: (i) no treatment (untreated); (ii) Cu[Dp44mT] (30 *μ*M); or (iii) Cu[DpC] (15 *μ*M). Cells were then stained for 12 min/37 °C with acridine orange (AO; 5 *μ*M). The AO staining was quantified as red fluorescence intensity/cell using ImageJ software. ***P<0.001 relative to the control (no treatment). ^###^*P<*0.001, relative to the respective non-U18666A-treated control. Scale bar=10 *μ*m. Photographs are typical of three experiments. Quantitation is mean±S.D. (three experiments)

Under control conditions, KBV1 (+Pgp) cells showed the distinct punctate AO red pattern (Figure 5ai), indicating intact lysosomes.^38^ Treatment with Cu[Dp44mT] (30 *μ*M/30 min/37 °C) or Cu[DpC] (15 *μ*M/30 min/37 °C) markedly and significantly (*P<*0.001) decreased the punctate red AO staining (Figures 5aii and aiii), which is consistent with LMP. The addition of U18666A alone to KBV1 cells (Figure 5bi) slightly increased (*P>*0.05) red AO fluorescence compared with untreated control cells (Figure 5ai). This effect may be explained by an increase in lysosome size.^35^ In clear contrast to control cells treated with Cu[Dp44mT] or Cu[DpC] (Figure 5ai, cf. with 5aii and 5aiii), there was no significant (*P>*0.05) change in punctate red AO staining after incubation with Cu[Dp44mT] (Figure 5bii) or Cu[DpC] (Figure 5biii) in U18666A-treated KBV1 cells, compared with the U18666A control (Figure 5bi).

In contrast to KBV1 cells, when assessing KB31 cells that express extremely low levels of Pgp^24^ (Figure 2ai), there was no significant (*P>*0.05) change in red AO staining in the presence or absence of U18666A after Cu[Dp44mT] or Cu[DpC] treatment (Figures 5cii, ciii, dii and diii) compared with untreated control cells (Figures 5ci and di). This observation suggests Pgp is important for Cu[Dp44mT]- and Cu[DpC]-induced LMP.

**Figure 6:**
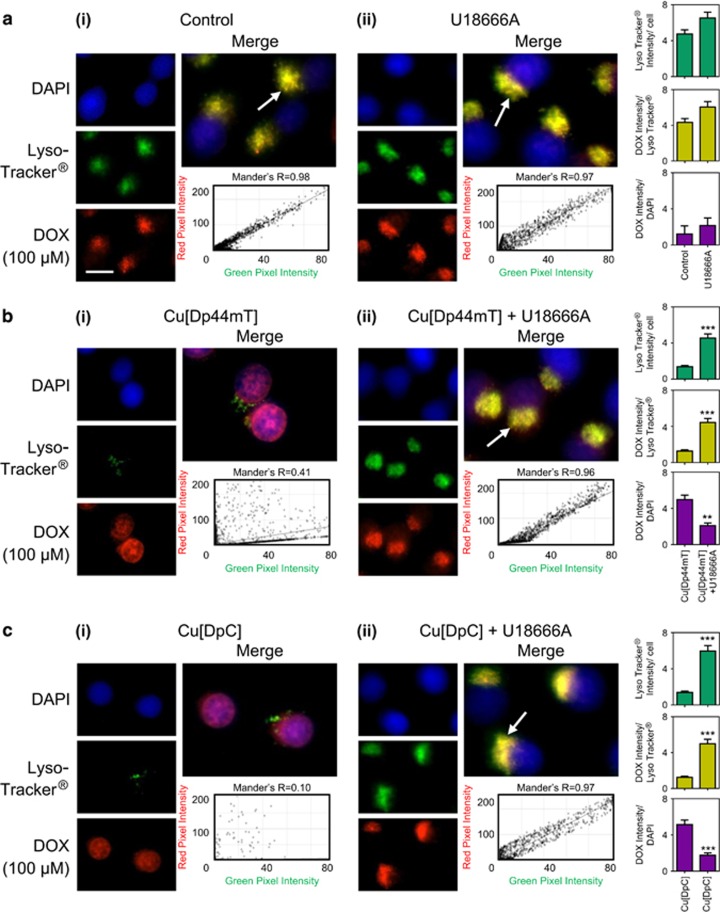
The cholesterol transport inhibitor, U18666A, prevents the release of DOX from LysoTracker Green-stained lysosomes following thiosemicarbazone treatment. Immunofluorescence microscopy images of KBV1 (+Pgp) cells treated with DOX (2 h/37 °C; 100 *μ*M), LysoTracker Green (40 min/37 °C; 100 nM) and 30 min/37 °C after either: (**a**) no treatment (Control), (**b**) Cu[Dp44mT] (30 *μ*M) or (**c**) Cu[DpC] (15 *μ*M), in the (i) absence of U18666A or (ii) presence of U18666A (2.3 *μ*g/ml). The overlap between DOX and LysoTracker Green (yellow merge; **a**–**c**) is indicated by Mander's overlap coefficient (*R*). LysoTracker Green was quantified as fluorescence intensity/cell, while DOX was quantified as DOX fluorescence intensity co-distribution with LysoTracker Green or DAPI, using ImageJ software. **P<0.001, ***P<0.001 relative to respective non-U16666A-treated control. Scale bar=10 *μ*m. White arrow=overlap between LysoTracker Green and DOX. Results in photographs are typical of three experiments. Quantitation is mean±S.D. (three experiments)

The studies herein using KBV1 cells with Cu[Dp44mT] and Cu[DpC] confirm and extend our previous investigation using Dp44mT, which demonstrated the ability of cholesterol loading with U18666A to prevent LMP by this thiosemicarbazone.^14^ Considering this, we next examined if U18666A could prevent Cu[Dp44mT]- and Cu[DpC]-induced redistribution of DOX from lysosomes to nuclei in KBV1 (+Pgp) cells (Figure 6).

Relative to the control where DOX and Lysotracker Green were strongly co-localized (*R*=0.98) in lysosomes (yellow-staining in the merge; Figure 6ai), Cu[Dp44mT] and Cu[DpC] treatment led to a pronounced decrease in LysoTracker Green staining and co-localization with DOX, and redistribution of DOX to nuclei (Figures 6bi and ci). In contrast, U18666A prevented the decrease in LysoTracker Green staining observed with Cu[Dp44mT] or Cu[DpC] treatment (cf. Figure 6bii with bi and cii with ci). This was consistent with Figures 5bi–biii, demonstrating that U18666A prevents lysosomal destabilization by thiosemicarbazones relative to control cells (Figures 5ai–aiii). Moreover, DOX-staining remained highly correlated with LysoTracker Green-stained lysosomes in the presence of U18666A (Cu[Dp44mT] *R*=0.96, Figure 6bii; Cu[DpC] *R*=0.97, Figure 6cii). This observation suggested U18666A stabilized lysosomes and prevented LMP by Cu[Dp44mT] or Cu[DpC].

Collectively, these results indicate U18666A decreases LMP caused by thiosemicarbazones, thereby preventing thiosemicarbazone-induced DOX redistribution to nuclei in Pgp-expressing cells.

### Drug synergy between DOX and Dp44mT or DpC can be prevented by stabilization of lysosomes with U18666A

**Figure 7:**
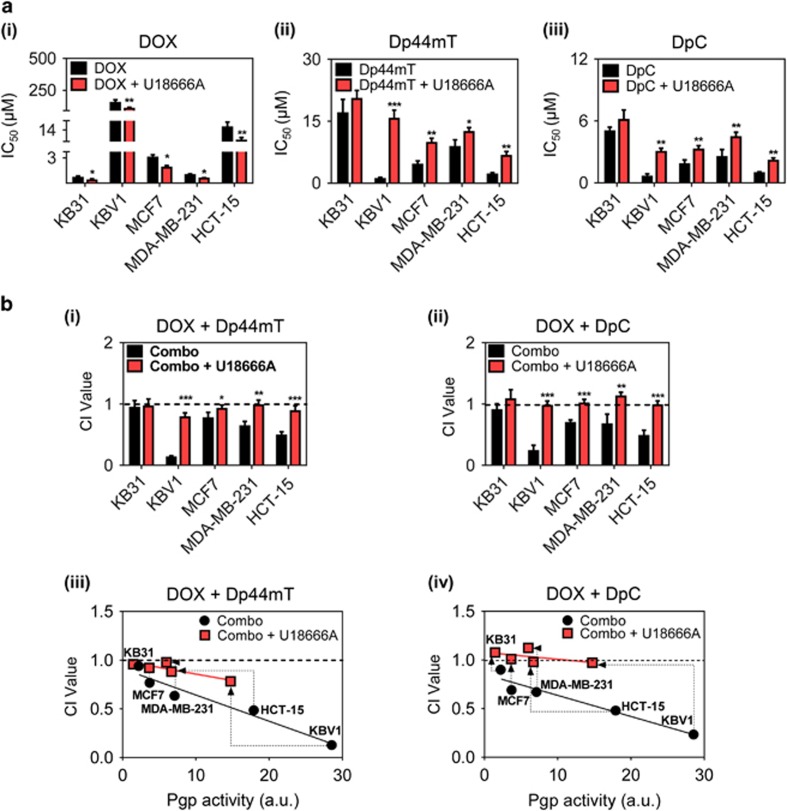
Inhibition of intracellular cholesterol transport by U18666A prevents drug synergy between DOX and the thiosemicarbazones, Dp44mT and DpC. (**a**) Cytotoxicity assays with the treatments: (i) DOX (72 h/37 °C), (ii) Dp44mT (24 h/37 °C) and (iii) DpC (24 h/37 °C), in the absence or presence of U18666A (2.3 *μ*g/ml). (**b**) Combination index (CI) values of the drug combination between: (i) DOX (72 h/37 °C) and Dp44mT (24 h/37 °C); or (ii) DOX (72 h/37 °C) and DpC (24 h/37 °C), as measured by the Chou-Talalay method.^25, 29^ The correlation between the CI values (**b**iii and biv) and the cellular Pgp activity (as measured by Rh123 retention) was plotted using linear regression for: (**b**iii) DOX (72 h/37 °C) and Dp44mT (24 h/37 °C); or (**b**iv) DOX (72 h/37 °C) and DpC (24 h/37 °C). CI>1 Antagonism, CI=1 Additive, CI<1 Synergistic, *P<0.05, **P<0.01, ***P<0.001 relative to the respective non-U18666A-treated control. (Black=Combo; red=Combo+U18666A). Dotted arrows indicate the alteration in CI value upon U18666A treatment. Results in (**a**) and (**b**) are mean±S.D. (three experiments)

Next, we examined if an increase in cholesterol and stabilization of lysosomal membranes using U18666A^14, 37^ could prevent synergism between DOX and thiosemicarbazones (Figure 2b). First, all cell types were examined for cytotoxicity to DOX (72 h/37 °C), Dp44mT (24 h/37 °C) or DpC (24 h/37 °C) in the absence or presence of U18666A (Figures 7ai–aiii). In all cells, DOX was slightly, but significantly (*P<*0.01–0.05), more cytotoxic in the presence of U18666A (Figure 7ai). In contrast to DOX, U18666A significantly (*P<*0.001–0.05) decreased the cytotoxicity of Dp44mT and DpC in all Pgp-expressing cell types (Figures 7aii and aiii). The greatest effect was a 15- and 5-fold decrease in sensitivity to Dp44mT and DpC, respectively, in KBV1 cells (Figures 7aii and aiii). There was a slight, but not significant (*P>*0.05) decrease in the sensitivity of KB31 cells to Dp44mT and DpC when treated with U18666A (Figures 7aii and aiii).

The Chou-Talalay method^25^ was then used to assess the synergistic interaction between DOX and Dp44mT or DpC in the presence of U18666A (Figures 7bi and bii). In all Pgp-expressing cells, U18666A significantly (*P<*0.001–0.05) decreased drug synergy between DOX and Dp44mT (Figure 7bi), as well as DOX and DpC (Figure 7bii). This decrease was most notable in KBV1 (+Pgp) cells, which had the greatest synergistic interaction under control conditions for both combinations. In KB31 cells, U18666A did not mediate a significant (*P>*0.05) change to CI values for either combination.

Considering that U18666A prevents LMP (Figures 5 and 6) by stabilizing the lysosomal membrane through cholesterol loading,^14, 36, 37^ we next examined the synergistic interaction between DOX and Dp44mT or DpC in the presence of U18666A. This was assessed by plotting the CI value with Pgp activity (measured by Rh123 retention) for each cell type (Figures 7biii and biv). Interestingly, U18666A partially decreased Pgp activity (see dotted arrows; Figures 7biii and biv) for cells with the highest Pgp level (i.e., KBV1, HCT-15 and MDA-MB-231) and also decreased synergy. These effects could be explained by (1) the known ability of U18666A to alter lipid membrane composition and cholesterol,^14, 36, 37^ which has been demonstrated to affect Pgp activity^40, 41, 42^ and (2) the ability of U18666A to prevent LMP (Figures 5 and 6). In fact, the partial decrease in Pgp activity observed with U18666A could not totally explain the pronounced reduction in synergy between thiosemicarbazones and DOX (Figures 7biii and biv). For example, while U18666A inhibited Pgp activity in KBV1 cells by ~50%, synergism between DOX and DpC was ablated, with the CI increasing from 0.23 to 0.97 (Figure 7biv). Collectively, these data indicate that both LMP and Pgp activity are important for synergism between thiosemicarbazones and DOX.

## Discussion

We investigated the interaction between DOX, which is more potent towards non-Pgp-expressing cells^4, 5, 6, 24^ and novel di-2-pyridylketone thiosemicarbazones, which preferentially target Pgp-expressing cells.^5, 6^ In these studies, DOX treatment led to a marked increase in Pgp (Figure 1d), which was attributed to drug-induced selection, allowing drug-resistant, Pgp-expressing populations to grow. In contrast to DOX, these studies identified that Dp44mT or DpC markedly decreased Pgp expression (Figure 1d), an effect due to the selective killing of Pgp-expressing cells (Figures 1c,3bii and biii) via lysosomal Pgp-mediated drug sequestration and LMP.^5, 6^ Consistent with this, Dp44mT and DpC displayed potent activity against Pgp-expressing tumor cells (Figures 3bii and biii).^5^ These observations form a strong rationale for combining these agents to target Pgp-expressing and non-Pgp-expressing cells.

Combination chemotherapy remains the mainstay treatment for tumors.^26^ However, there is a lack of information regarding the mechanistic interactions and drug redistribution when combining agents. Notably, Pgp has two roles in drug resistance: (1) drug efflux from cells,^3, 13, 43^ and (2) drug trapping within lysosomes, which acts as a drug safe house resulting in resistance.^4^ Both these effects could hinder treatment.^4, 44, 45^ Consequently, understanding the cellular distribution of chemotherapeutics, and how to manipulate this through combination treatments, is imperative for developing treatments against resistance.

**Figure 8:**
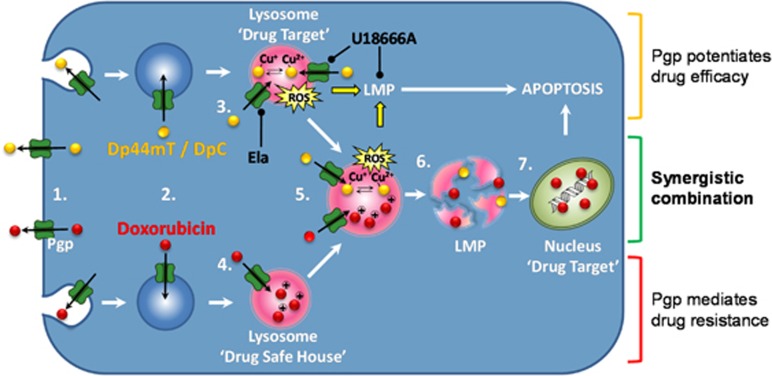
Schematic illustration of the synergistic interaction between Doxorubicin and Dp44mT/DpC. (1) Pgp on the plasma membrane actively pumps Pgp substrates out of cells. As part of endocytosis, the plasma membrane containing Pgp buds inwards to form early endosomes. (2) As a consequence of endocytosis, the topology of Pgp is inverted, and thus substrates are transported into the vesicle lumen. As the endosome matures into the lysosome, it becomes increasingly acidified. When a Pgp substrate, such as (3) Dp44mT/DpC, or (4) DOX, enters the cell, the drug is sequestered into the acidic lysosomes by Pgp-transport activity.^4, 5, 6^ If the substrate is protonated at acidic pH (such as DOX and Dp44mT/DpC), it becomes trapped in lysosomes.^4, 5, 6, 9^ The trapping of protonated drugs prevents substrates reaching their molecular targets (e.g., the nucleus for DOX).^4^ (3) However, once trapped in the lysosome, Dp44mT or DpC bind copper and redox cycle forming reactive oxygen species (ROS) that cause lysosomal-membrane permeabilization (LMP) and then apoptosis.^5, 6, 9, 16^ (5) When added in combination with DOX, Dp44mT or DpC redox cycle in lysosomes containing trapped DOX. (6) Dp44mT- or DpC-induced LMP causes the release of trapped DOX. (7) Then DOX redistributes to its molecular target, the nucleus. Notably, the Pgp-transport inhibitor, Ela, prevents entrance of Dp44mT or DpC into lysosomes, blocking LMP and the release of DOX to the nucleus. On the other hand, U18666A partially inhibits Pgp activity and also stabilizes the lysosomal membrane by cholesterol loading and prevents LMP mediated by Dp44mT or DpC, inhibiting lysosomal DOX release

The lysosomal trapping of chemotherapeutics, such as DOX, prevents their activity, resulting in drug resistance.^4^ Interestingly, thiosemicarbazones are also sequestered into lysosomes by Pgp, where they become charged and trapped in the acidic lysosomal pH.^5, 9, 15, 46^ However, the pharmacology of these agents differ from most cytotoxic drugs due to their ability to potently redox cycle with metals such as copper.^5, 9, 15, 18, 46^ It is this ability to markedly induce ROS that induces LMP and cell death.^4, 9^ Notably, our previous studies have demonstrated that control thiosemicarbazones (i.e., Dp2mT or Bp2mT) that cannot bind metal ions (including copper ions) do not possess antiproliferative activity or induce LMP.^7, 9, 16^ Herein, we report for the first time that Dp44mT and DpC when used in combination with DOX can cause redistribution of DOX from lysosomes to the nucleus (Figure 4). This effect overcomes resistance and results in markedly increased tumor cell susceptibility due to two potent cytotoxic effects. That is, thiosemicarbazone induced LMP, which results in pronounced cytotoxicity,^5, 6, 9^ and also DOX release from lysosomes, allowing its access to nuclear targets (Figures 4 and 8).

Using Chou and Talalay analysis,^29^ the combination of DOX and Dp44mT or DOX and DpC was synergistic in Pgp-expressing cell types (Figure 2b). In light of the correlation between Pgp activity and increased drug synergy between thiosemicarbazones and DOX, it was demonstrated the mechanisms facilitating thiosemicarbazone anti-cancer activity, namely Pgp activity and LMP,^4, 9, 46^ are crucial for drug synergy. Indeed, inhibiting Pgp with Ela decreased synergy in Pgp-expressing cells (Figures 3ci and cii). Notably, since only 16% of thiosemicarbazones entering lysosomes become trapped,^9^ it is clear the active transport process mediated by Pgp greatly increases the amount of the agent reaching the lysosome to enhance trapping.^5, 6^

Importantly, LMP was imperative for synergy, as U18666A, which increases lysosomal stability via cholesterol loading,^14, 37^ prevented thiosemicarbazone-induced LMP (Figure 5). This effect prevented DOX redistribution from lysosomes to nuclei (Figure 6). We attribute this effect to U18666A stabilizing lysosomal membranes against oxidative stress and also partially inhibiting Pgp activity (Figure 7aiii and aiv), thereby preventing LMP (Figure 8).^14, 35^ The ability of U18666A to inhibit LMP by Dp44mT or DpC prevents DOX release from lysosomes and avoids nuclear targeting (Figure 6), inhibiting its cytotoxicity (Figure 7b). Hence, through U18666A partially inhibiting Pgp activity and preventing Dp44mT- and DpC-mediated LMP, which itself is cytotoxic, this inhibits DOX release from lysosomes and prevents synergism between these agents (Figure 8).

In conclusion, the mechanisms behind resistance to many chemotherapeutics are still largely unknown, and there is an urgent need to develop rational therapeutic regimens circumventing resistance. This study demonstrates the efficacy of DOX could be markedly enhanced by DpC or Dp44mT, which permeabilize lysosomes via Pgp to enable DOX release and its nuclear targeting (Figure 8). This synergy allows the use of significantly less of each these agents to obtain the same tumor cell kill, and as such, also minimizes toxicity to normal tissues.

## Materials and Methods

### Chemicals

DOX was purchased from Pfizer (New York, NY, USA). 3-(4,5-Dimethyl-2-thiazolyl)-2,5-diphenyl-2H-tetrazolium bromide (MTT), 3-*β*-(2-(diethyl-amino)ethoxy)androst-5-en-17-one (U18666A), vinblastine (VBL), Rhodamine-123 (Rh123) and Elacridar (Ela; PSC833) were purchased from Sigma-Aldrich (St. Louis, MO, USA). Both Dp44mT and DpC, as well as their copper complexes (i.e., Cu[Dp44mT] and Cu[DpC]), were synthesized and characterized, as described previously.^11, 16, 17, 18^

### Cell culture

Cell types were purchased from the American Type Culture Collection (ATCC, Manassas, VA, USA). The cervical carcinoma, KBV1 and KB31 cell lines, were maintained in DMEM media. Moreover, medium for growing KBV1 cells was supplemented with VBL (1 *μ*g/ml) to maintain a full resistance phenotype.^19^ The colorectal HCT-15 cell line was grown in RPMI media, and the breast cancer cell lines, MCF7 and MDA-MB-231, were maintained in MEM media (media were from Life Technologies, Carlsbad, CA, USA). All media were supplemented with 10% fetal bovine serum, penicillin (100 U/ml), streptomycin (100 mg/ml), glutamine (2 mM), non-essential amino acids (1 mM) and pyruvate (1 mM; all from Life Technologies). Unless otherwise stated, the Pgp inhibitor, Elacridar (Ela; Sigma-Aldrich; 0.2 *μ*M), was added for 30 min/37 °C prior to drug treatments and remained for the duration of the experiments. The cholesterol transport inhibitor, U18666A (2.3 *μ*g/ml; Sigma-Aldrich), was added for 48 h/37 °C prior to, and then also during drug treatments.

### Proliferation/viability assays

Cellular proliferation was assessed using MTT assays, which were validated by viable cell counts using standard procedures.^20^ Cells were incubated with serial dilutions of DOX for 72 h/37 °C or thiosemicarbazones (Dp44mT, DpC) for 24 h/37 °C.

### Protein extraction and western blotting

Protein extractions and western blotting were performed using standard procedures.^21, 22^ Membranes were probed using mouse anti-human Pgp (Cat. #:P7965; 1:5000; Sigma-Aldrich) or mouse anti-*β*-actin (Cat. #:A5441; 1:5000; Sigma-Aldrich), which was used as a protein-loading control. Incubations were performed overnight/4 °C followed by incubation with a secondary antibody (1 h/RT), namely horseradish peroxidase-conjugated goat anti-mouse (Cat. #:A4416; 1:10 000, Sigma-Aldrich).

### Rh123-accumulation assay

Pgp functionality was assessed by measuring intracellular accumulation of the fluorescent Pgp substrate, Rh123.^23^ Following treatment, cells were incubated with Rh123 (10 *μ*M; 30 min/37 °C) and then analyzed by flow cytometry (FACSCanto II; BD Biosciences, San Jose, CA, USA) at 510 nm excitation/595 nm emission. Data were collected using 10 000 cells/sample.

### Chou-Talalay combination analysis

Combinations of DOX (72 h/37 °C) with Dp44mT (24 h/37 °C) or DpC (24 h/37 °C) were assessed according to general methods.^11^ Briefly, as per standard procedure in calculating combination index (CI) values, cells were incubated with concentrations of DOX in a range that was between eight times below and eight times above the IC_50_ value of DOX after a 72 h/37 °C incubation (i.e., 0.125, 0.25, 0.5, 1, 2, 4, 8 × IC_50_). Clearly, the concentrations of DOX used were cell line dependent, as each cell type had it own individual IC_50_ value for this chemotherapeutic. For example, using KB31 cells (extremely low Pgp levels)^24^ that were highly sensitive to DOX, the concentration of this agent ranged from 0.05 to 3.4 *μ*M, while in resistant KBV1 (+Pgp) cells, the DOX concentration used ranged between 18 and 1200 *μ*M.

As for DOX, to calculate the CI values, the thiosemicarbazones, Dp44mT or DpC, were incubated with cells at the same concentration range above and below their IC_50_ values over a 24 h/37 °C incubation (i.e., 0.125, 0.25, 0.5, 1, 2, 4, 8 × IC_50_). Again, the concentrations of Dp44mT or DpC implemented were cell type dependent. For instance, with KB31 cells (extremely low Pgp levels)^24^ that were less sensitive to thiosemicarbazones, the concentration of these agents ranged from 2.1 to 135 *μ*M (Dp44mT) or from 0.7 to 46 *μ*M (DpC). In contrast, for KBV1 (+Pgp) cells that were more sensitive to these thiosemicarbazones, the concentrations implemented ranged from 0.2 to 12 *μ*M (Dp44mT) or from 0.07 to 5 *μ*M (DpC). The resulting dose–response curves were then analyzed using the methodology of Chou-Talalay.^25^

Notably, in combination studies with DOX and the thiosemicarbazones, DOX was present in the incubation for the entire 72 h/37 °C, while Dp44mT or DpC were present in the final 24 h of this incubation. The rationale behind this incubation sequence was to load lysosomes initially with DOX, followed by incubation with the thiosemicarbazones to induce lysosomal permeabilization and the release of DOX from the lysosomal compartment. The timing of this incubation sequence was demonstrated to be optimal in preliminary experiments to induce synergism between the drug combinations relative to incubating the agents together for the entire incubation. Hence, the initial loading of the lysosomal compartment with DOX, followed by permeabilization with thiosemicarbazones, was required to obtain synergy. Notably, studies utilizing co-incubation of thiosemicarbazones (72 h) with DOX (72 h) were less optimal in terms of synergy, and hence were not examined further.

### Transient Pgp silencing using siRNA

Cellular Pgp expression was silenced using two separate *MDR1* siRNAs (Cat.#:4123, #1; and 3933, #2; Ambion, Carlsbad, CA, USA). Briefly, the siRNA-Lipofectamine mixture (50 nM *MDR1* siRNA and 1:400 Lipofectamine 2000; Life Technologies) was added to the cells (at 30% confluency), and incubated for 72 h/37 °C prior to further experimentation.^4, 5^ The effectiveness of Pgp-silencing was examined using western blotting, Rh123 retention assays and MTT cytotoxicity assays. A negative control siRNA (NC siRNA; Cat. #:AM4635; Thermo Fisher Scientific, Waltham, MA, USA) was used at the same concentration as *MDR1* siRNA.

### Lysosomal staining for assessment of the uptake of DOX

LysoTracker Green staining (Life Technologies) of lysosomes was visually assessed by fluorescence microscopy. Cells were incubated with DOX (100 *μ*M) for a total of 2 h/37 °C. LysoTracker Green (100 nM) and DAPI nuclear stain (0.5 *μ*M; Invitrogen) were then added for the last 40 min/37 °C. Finally, Cu[Dp44mT] (25 *μ*M) or Cu[DpC] (10 *μ*M) was added for the final 30 min/37 °C. Live cells were visualized for green (495 nm excitation/516 nm emission), red (577 nm excitation/592 nm emission) and blue (358 nm excitation/461 nm emission) fluorescence (Axio Observer Z1 microscope; Zeiss, Oberkochen, Germany) using a LD Plan-NEOFLUAR 40x/0.6 Ph2 Korr objective.

### Assessment of lysosomal membrane permeability

The lysosomotropic stain, AO (Sigma-Aldrich), was used to determine LMP.^26, 27^ Cells were stained with AO (2.5 *μ*g/ml; 12 min/37 °C), then washed twice with media and incubated with the copper complexes of Dp44mT (Cu[Dp44mT] 25 *μ*M) or DpC (Cu[DpC] 10 *μ*M) for 30 min/37 °C. Live cells were visualized in phenol-red-free DMEM media (Life Technologies) for green (495 nm excitation/516 nm emission) and red (577 nm excitation/592 nm emission) fluorescence using the Axio Observer.Z1 microscope and objective described above.

### Data analysis

Results are shown as mean±S.D. (*n*=3 experiments). Statistical analysis was performed using a Student's *t*-test or one-way analysis of variance in Prism 6.0 (Graphpad Software, San Diego, CA, USA). Data was considered statistically significant when *P<*0.05. The concentration–response curves data were fitted using Prism 6.0 (Graphpad Software) to obtain IC_50_ values. Fluorescence intensity and Mander's overlap coefficient for image co-distribution were estimated using ImageJ 4.7v software (National Institutes of Health, Baltimore, MD, USA). Flow cytometry data analysis was performed using FlowJo software (FlowJo LLC, Ashland, OR, USA). Western blot densitometry was performed using ImageLab Software (Bio-RAD, Hercules, CA, USA). Combination data were assessed using CalcuSyn Software (Biosoft, Cambridge, UK), according to the Chou-Talalay method.^25^ The slopes and elevations of the linear regression graphs were compared between two treatment groups using Prism 6.0 (Graphpad Software). Pgp activity was measured as the inverse correlation of Rh123 accumulation in cells.
